# Recent progress in advanced biomaterials for long-acting reversible contraception

**DOI:** 10.1186/s12951-022-01329-5

**Published:** 2022-03-17

**Authors:** Mingzhe Yan, Yanming Zhang, Zhihang Wu, Yifei Li, Keke Dou, Banghui Wang, Yingruo Wang, Qihui Zhou

**Affiliations:** 1 Institute for Translational Medicine, Department of Stomatology, The Affiliated Hospital of Qingdao University, Qingdao University, Qingdao, 266003 China; 2 Department of Human Anatomy, Histology and Embryology, School of Basic Medicine, Qingdao University, Qingdao, 266073 China; 3 Department of Biomedical Engineering, The University of Melbourne, Victoria, 3010 Australia; 4 Shandong University of Science and Technology, Qingdao, 266590 China

**Keywords:** Long-acting reversible contraception, Biomaterials, Biodegradable implant, Delivery route, Controlled drug delivery

## Abstract

Unintended pregnancy is a global issue with serious ramifications for women, their families, and society, including abortion, infertility, and maternal death. Although existing contraceptive strategies have been widely used in people's lives, there have not been satisfactory feedbacks due to low contraceptive efficacy and related side effects (e.g., decreased sexuality, menstrual cycle disorder, and even lifelong infertility). In recent years, biomaterials-based long-acting reversible contraception has received increasing attention from the viewpoint of fundamental research and practical applications mainly owing to improved delivery routes and controlled drug delivery. This review summarizes recent progress in advanced biomaterials for long-acting reversible contraception via various delivery routes, including subcutaneous implant, transdermal patch, oral administration, vaginal ring, intrauterine device, fallopian tube occlusion, vas deferens contraception, and Intravenous administration. In addition, biomaterials, especially nanomaterials, still need to be improved and prospects for the future in contraception are mentioned.

**Graphical Abstract:**

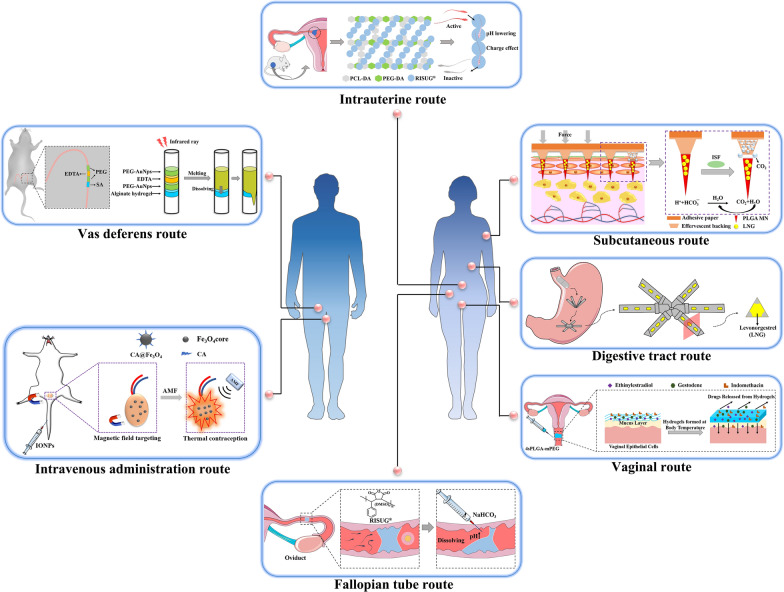

## Introduction

The importance of sexual life to human health and the overall well-being of society has been increasingly recognized. Proper sex life not only relieves individual stress and maintains family harmony, but also maintains the female reproductive organ health [1]. However, unintended pregnancy is a global issue with serious ramifications for women, their families, and society, including abortion, infertility, and maternal death. There are approximately 85 million unwanted pregnancies and 40–60 million abortions worldwide each year [2]. Abortion inevitably damages the female endometrium, which can cause menstrual disorder, uterine adhesion, intrauterine infection, habitual abortion, and even lifelong infertility [3]. Although existing contraceptive strategies have been widely used in people’s lives, there has not been much satisfactory feedback. Long-term oral hormonal contraceptives can cause endocrine disorder, impaired glucose tolerance, abnormal liver function, and even an increased risk of thrombotic disease [4, 5]. The improper use of condoms can lead to contraceptive failure, and allergic symptoms may be caused by the application of latex [6]. According to the report from the World Health Organization (WHO) to investigate the effectiveness of contraceptive methods in 2020, traditional therapies e.g., sperm–egg fusion disruption alone, emergency drug contraception, and standard date calculation are all not effective in contraception (Table 1) [7]. In addition, although both male and female sterilization have low rates of re-pregnancy, they do not meet the requirements of post-contraceptive reproduction and protection against the risk of future child demise. Therefore, the design and development of long-acting reversible contraception are emerging as a promising strategy to address the issues above.

**Table 1:** Effectiveness of contraceptive methods [7]

Method	Effectiveness	Method	Effectiveness
Female condoms	5	Female sterilization (tubal ligation)	0.5
Standard days method	5	Combined oral contraceptives	0.3
TwoDay method	4	Progestogen-only pills	0.3
Withdrawal	4	Combined contraceptive patch and combined contraceptive vaginal ring	0.3
Male condoms	2	Progestogen-only injectables	0.2
Emergency contraception pills	2	Implants	0.1
Sympto-thermal method	1	Male sterilization (vasectomy)	0.1
Lactational amenorrhea method	0.9	Monthly injectables or combined injectable contraceptives	0.05
Intrauterine device (IUD): copper-containing	0.6	Basal body temperature method	None
Intrauterine device (IUD) levonorgestrel	0.5	Calendar method or rhythm method	None

Effectiveness: pregnancies per 100 women per year with consistent and correct use. None: reliable effectiveness rates are not available

**Fig. 1:**
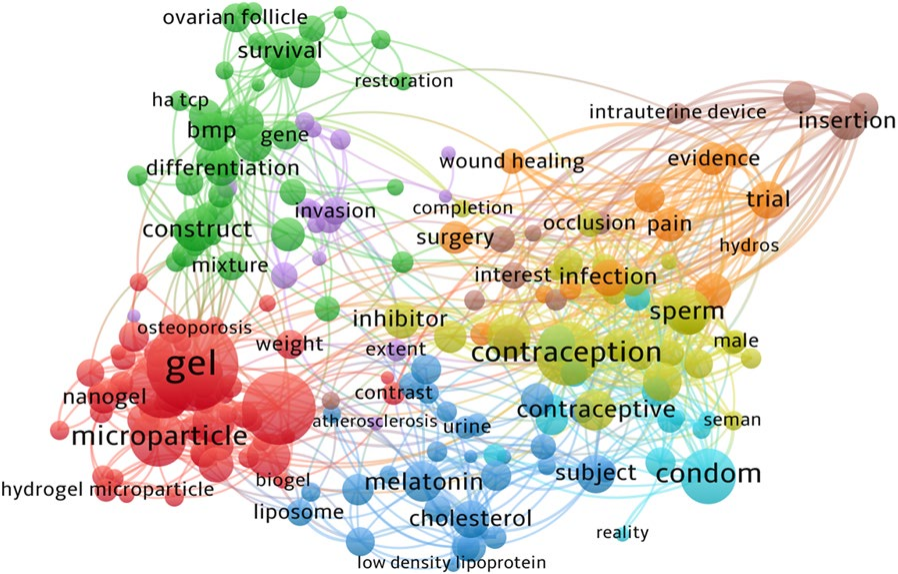
The analysis of keyword co-occurrences on biomaterials and contraception

In recent years, biomaterials-based long-acting reversible contraception has received increasing attention from the viewpoint of fundamental research and practical applications mainly owing to improved delivery routes and controlled drug delivery [8–10]. “Biomaterials are those materials—be it natural or synthetic, alive or lifeless, and usually made of multiple components—that has been engineered to interact with biological systems for a medical purpose—either a therapeutic (treat, augment, repair, or replace a tissue function of the body) or a diagnostic one.” [11, 12]. As shown in Table 2, various types of biomaterials have been used in a wide range of contraceptives based on their characteristics and superiorities. And the report of scientific researches showed that biomaterials are widely studied in the field of contraception in the form of delivery carriers compared to oral or injection administration (Fig. 1). Biomaterials with various components and architectures provide visible and tangible reactions to modulate tissue microenvironment for practical applications [13–22]. Particularly, for long-acting reversible contraceptives, biomaterials can not only serve as an effective drug or hormonal carrier for long-term stable release in vivo but also respond to conditioned stimuli in the body, solving problems such as peristalsis causing the device to slip. In addition, the unique (bio)physicochemical properties of biomaterials including spermicidal action, stable degradability, and stiffness, can also be used directly or as an adjunct to long-acting contraception. Also, the controllable biodegradability, photothermal and chemical responses of the advanced biomaterials can be used to achieve a reversible recovery of fertility with minimal or no damage.

**Table 2:** Biomaterials used in contraception

Type	Characteristics	Examples	Products	Defects	Ref.
Metallic biomaterials	Spermicidal effect and antibacterial properties	Cu (Cu^2+^), Iron oxide	Cu-IUD, nanoparticles	Biological toxicity of oxidation products	[23, 24]
Polymeric biomaterials	Adequate mechanical and physical properties, bio-inertness, and good biocompatibility	Latex, silicone, PE	Condoms, implants, IUD	Non-biodegradability and poor drug-delivery efficiency	[25–27]
Composite biomaterials	Bioactive interaction and plasticity	SMA, PLGA, GDL	RISUG^®^, Vaginal gel	Potential foreign body reactions	[28, 29]
Biodegradable biomaterials	Nature source, good biodegradability, and biocompatibility	CS, β-GP	Microneedle patch	Difficulty to obtain the high purity materials	[30]

Cu: Copper; Cu^2+^: Copper ion; Cu-IUD: Copper-containing IUD; PE: polyethylene; IUD: intrauterine device; SMA: styrene maleic anhydride; PLGA: polylactic acid co-glycolic acid; GDL: gluconolactone; CS: chitosan; β-GP: β-sodium glycerophosphate

**Fig. 2:**
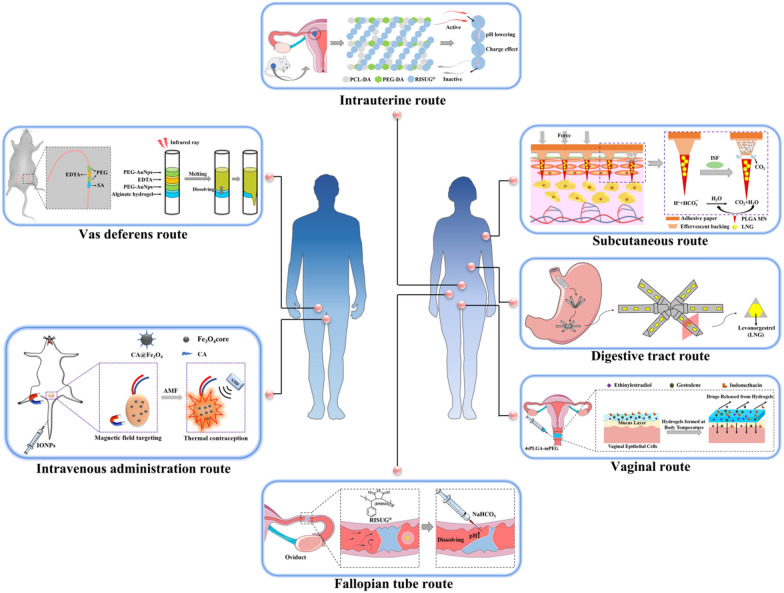
The schematic illustration of advanced biomaterials applied in contraception by various routes

In this review, we summarized recent advances in biomaterials-based long-acting reversible contraception via various delivery routes, including subcutaneous implant, transdermal patch, oral administration, vaginal ring, intrauterine device, fallopian tube occlusion, vas deferens contraception, and intravenous administration (Fig. 2). In addition, biomaterials, especially nanomaterials, still need to be improved and prospects for the future in contraception are mentioned.

## Reproductive system and fertilization process

The reproductive system refers to the tissues, glands, and organs required for the reproduction of human offspring. Regulated by the neuro-endocrine system, the system is responsible for the maturation of sperm and eggs, fertilization, pregnancy, and childbirth [31]. The main contraceptive methods intervene in the early stages of the pregnancy by inhibiting the secretion of hormones or cytokines, blocking pathways, and altering the environment of implantation in the uterus, which prevents the maturation of the sperm and egg, cuts off the sperm–egg fusion pathway and interfere with the implantation of the fertilized egg [32].

### Male reproductive system and spermatogenesis

A male reproductive system is a group of organs that can promote sperm maturation, storage, and ejaculation, as well as secrete androgens for development and maintain androgynous characteristics [33]. It consists mainly of the gonads (testis), the vas deferens (epididymis, vas deferens, ejaculatory ducts, and urethra), the accessory glands (seminal vesicle glands, prostate gland, urethral bulb glands), the scrotum, and the penis.

**Fig. 3:**
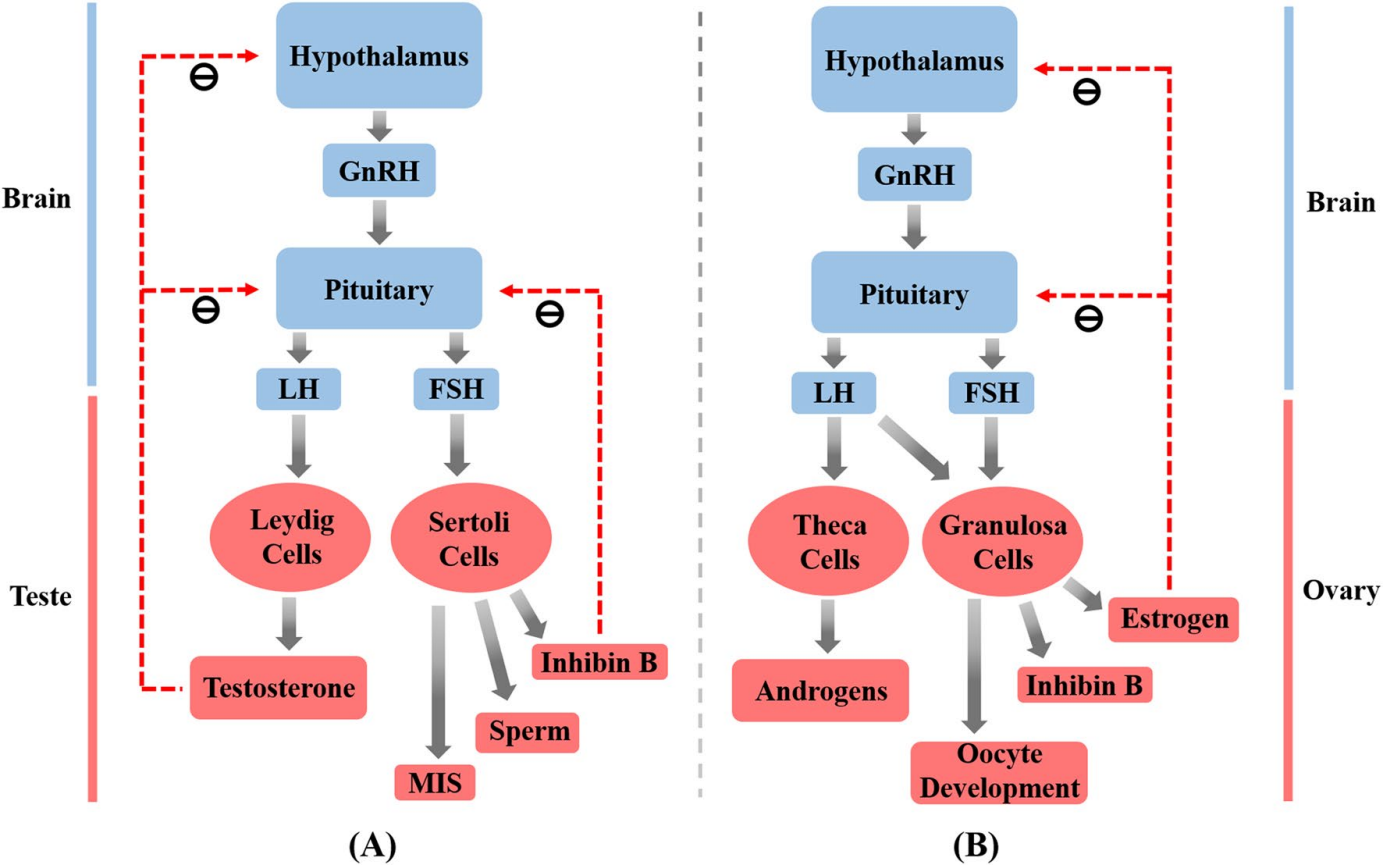
**A** The male hypothalamic–pituitary–gonadal axis. **B** The female hypothalamic–pituitary–gonadal axis

The testis is the main gland of spermatogenesis and androgen secretion, which are regulated by the hypothalamic–pituitary–gonadal axis (Fig. 3A) [34]. The hypothalamus secretes gonadotropin-releasing hormone (GnRH) into the hypothalamic–pituitary portal system, stimulating the anterior pituitary gland, which further promotes the release of luteinizing hormone (LH) and follicle-stimulating hormone (FSH). LH stimulates the secretion of carbon chain enzymes from testicular interstitial cells to convert cholesterol into testosterone, and FSH stimulates testicular supporting cells to promote spermatogenesis as well as release inhibin B and Mullerian inhibiting substances (MIS) hormone. Testosterone is essential for maintaining a normal libido and sexual function, promoting the maturation of the sexual organs, and maintaining the secondary sexual characteristics of the male.

The primitive spermatocytes are located in the periphery of the spermatogenic tubules and the supporting cells activated by FSH promote spermatogenesis. During 13 days, the spermatogonia complete two meiotic divisions to form haploid spermatocytes, which are simultaneously released into the epididymis for maturation and storage in the caudal epididymis until ejaculation occurs. On the other hand, testosterone reduces the secretion of LH and FSH through negative feedback from the hypothalamus and anterior pituitary. Inhibin B reduces the release of FSH by a similar negative feedback mechanism.

During sexual arousal, the penis is engorged with blood causing penis erection. With the sexual stimulation continuing, the smooth muscle of the epididymis contracts to push sperm into the vas deferens, which are located in the spermatic cord. The vas deferens are connected to the seminiferous ducts near the prostate and carry the sperm to the ejaculatory ducts to reach the female vagina.

### Female reproductive system and fertilization

The female reproductive system plays a critical role in ovulation, fertilization, pregnancy, childbirth, promoting growth, as well as developing and maintaining female secondary sexual characteristics [35]. It consists mainly of the internal genitalia, including the vagina, uterus, fallopian tubes and ovaries, and the vulva. Unlike the male reproductive system, the female reproductive system has a regular menstrual cycle and ovulation regulated by the hypothalamic–pituitary–gonadal axis (Fig. 3B).

The menstrual cycle refers to the cyclical changes in the functional layer of the endometrium under the cyclical action of estrogen and androgens secreted by the ovary from puberty, and the process of endometrial exfoliation, bleeding, repair and proliferation occurs every 28 days, including the follicular phase, ovulation phase, and luteal phase. The ovulation period generally occurs approximately 14 days before the next menstrual cramp. Similarly, GnRH secreted by the hypothalamus stimulates the anterior pituitary to release FSH and LH. Under the stimulation of FSH, the granulosa cells in the ovary secrete estradiol (E2) and inhibin, and the negative feedback regulates the secretion of the hypothalamus and pituitary gland so that the level of FSH gradually decreases. LH acts on the surface receptors of the follicular membrane cells to promote follicular development and secrete androgens. In the late follicular phase, with the E2 level continuing to rise, it produces a positive feedback effect on the hypothalamus and pituitary, which causes the LH level to rise sharply, and ovulation occurs. Following the releasing its ovum, the follicle transforms into a corpus luteum and secretes progesterone, 17β-progesterone, oestradiol, androstenedione, etc. Progesterone stimulates the transformation of the endometrium from proliferative to secretory, which involves the secretion of chemokines, growth factors, and cell adhesion molecules, the thickening of the endometrium, and the distribution of spiral arteries that facilitate the implantation of the zygote.

The sperm and egg fusion in the abdomen of the fallopian tube and complete the fertilization process to form a zygote. The zygote remains in the fallopian tube for 3 days and undergoes a series of cell divisions, differentiation, and eventually the formation of a mulberry embryo. The mulberry embryo moves along the fallopian tube towards the horn of the uterus and enters the uterus in the form of an embryo. Over approximately 6 days, the zygote develops into a blastocyst and completes its implantation in the endometrium, while actively secreting human chorionic gonadotropin (hCG), which allows the corpus luteum to continue to produce progesterone, thus maintaining the thickened endometrium for the continuation of the pregnancy.

## Advanced biomaterials for contraception by various routes

The choice of contraceptive method has always been a focus of concern, with safety, effectiveness, availability (including accessibility and affordability) and acceptability being factors often considered [36]. It is noted that no form is 100% effective, but certain methods can be particularly effective through contraceptive mechanisms. In addition to preventing pregnancy, some contraceptive methods also have effects like preventing sexually transmitted infections and antibacterial properties [37]. The frequently used methods have been listed along with their mechanisms and pros and cons analysis, as shown in Table 3. It has been demonstrated that biomaterials with unique (bio)physicochemical characteristics can not only improve the effectiveness and safety of contraceptive methods through a series of routes, but also focus on exploring post-contraceptive reversibility and allowing long-acting reversible contraception.

**Table 3:** The frequent methods for contraception [38–40]

Method	Device	Mechanism	Pros and cons
Hormonal methods	Implant	The implant is a progestin-coated silicone rod that is implanted in the woman’s upper arm by the medical professional. The progesterone in the silicone rod being released slowly prevents normal ovulation and thickens the mucus at the entrance to the uterus so that sperm cannot pass through	The implant has a high contraceptive efficiency for up to 3 years. It does not disturb sexual life during this period and allows a rapid recovery of fertility after removal. However, its earlier use may cause a reduction in menstrual blood flow or even amenorrhoea, as well as a localized sensation of abnormality on insertion, which requires professional handling. Caution should be taken in patients with severe liver disease and those at risk of blood clots
	Patch	The patch is attached to the lower abdomen, buttocks, or upper body, where the oestrogen and progesterone are absorbed into the body through the skin, inhibiting the production of FSH and LH, thus preventing normal ovulation as well as thickening the mucus secreted at the entrance to the uterus, thus making it more difficult for sperm to pass	Compared to the implant, the patch is easy to operate and does not require professional assistance, while the fertility is recovered more quickly. Performed once a week, but with a lower contraceptive efficiency
	Injector	The synthetic depot medroxyprogesterone acetate (DMPA) is used to achieve contraception by intramuscular injection, preventing normal ovulation as well as thickening the mucus secreted at the entrance to the uterus, thus making it more difficult for sperm to pass	DMPA has a remarkable contraceptive efficacy and can be administered to relieve menstrual pain and prevent diseases such as ovarian and endometrial cancers. However, patients need to be injected every three months and may experience progesterone-like symptoms with initial use. Secondly, the recovery of fertility needs to wait for 18 months after stopping use. Caution should be taken in patients at risk of blood clots
	Progestin-only pill	The progestogen-only pill contains the hormone progesterone, which thickens the mucus at the entrance to the uterus and alters the endometrium, preventing the entry of sperm into the uterus and fertilization	The progestogen-only pill is suitable for most women, but needs to be taken every day and may cause progestogen-like symptoms on initial use
	Combined oral contraceptives	The combination of estrogen and progestin administered orally can prevent normal ovulation and also makes the mucus secretion from the uterine entrance sticky, more difficult for sperm to pass through	The contraceptive is highly effective when taken correctly and the combined oral administration for suitable age up to 50 years old facilitates to reduce painful menstrual periods. However, the initial use may cause strong progestin-like symptoms, e.g., breast tenderness, weight gain, reduced libido, especially in patients at risk of blood clots
	Vaginal ring	The vaginal ring is a plastic drug delivery platform for estrogen and progesterone that is placed in the vagina. The vaginal wall absorbs the released hormones, thereby inhibiting ovulation and controlling menstruation	The contraceptive effect may be not immediate and the ring is not easily fixed in the vagina, Besides, the pregnant and patients at risk of blood clots should be cautious to use it
Intrauterine contraception	Hormonal-IUD	The IUD is divided into hormonal-IUD and Cu-IUD. Hormonal-IUD consists of plastic and progestin (e.g. LNG), which is slowly released after it is inserted into the uterus by the medical professional. Hormones can thicken the mucus entering the uterine mouth, prevent sperm from passing through, and thin the uterine wall, making it difficult for fertilized eggs to implant. Copper IUD consists of plastic and copper. Copper is toxic to sperm and changes endometrium, making it difficult for fertilized eggs to implant	The IUD provides good contraceptive effectiveness, which can be effective for 5 to 10 years. The administration has no effect on normal sexual life and the rapid recovery of fertility after removal can be achieved. However, the initial use of the device may cause side effects such as menstrual pain, irregular bleeding, and breast pain
	Cu-IUD		
Barrier methods	Condom	The condom works as a physical barrier, worn on the penis or inserted into the vagina during sex, preventing body fluids from passing	It works well when used correctly and can prevent the spread of sexually transmitted diseases. Improper use or condom breakage can lead to unwanted pregnancy, and latex has allergic potential
	Spermicide	Spermicide in various forms (e.g. foam, gel, cream, film, suppository, or tablet) is placed in the vagina to kill sperm that are released into the vagina	Spermicide can be easily operated without significantly interrupting the sexual experience. However, it is poor contraceptive effectiveness
Fertility awareness		Fertility awareness refers to avoiding sexual intercourse during the time of the menstrual cycle when the woman is most fertile	No requirement for contraception during intercourse, but a higher rate of unwanted pregnancy than other methods
Withdrawal		The withdrawal refers to the removal of the penis from the vagina before ejaculation to prevent the release of sperm into the vagina	This method has a satisfying sexual experience, but has a higher rate of unwanted pregnancy because the male body fluid may still contain sperm before ejaculation
Lactational amenorrhea		A lactational amenorrhea method is a form of temporary contraception in which a woman who gives birth to the fetus does not have a menstrual period, meaning that ovulation does not occur	The method can be used only if three conditions are met: (1) less than 6 months after childbirth, (2) amenorrhea, and (3) fully breastfeeding
Sterilization	Tubal ligation	Sterilization is a medical treatment that permanently prevents pregnancy by blocking the pathway for ejaculation or sperm–egg binding through the vas deferens or tubal ligation	Sterilization provides permanent contraception with high safety and a low risk of unwanted pregnancy. However, it is relatively difficult or impossible to restore fertility after sterilization
	Vasectomy		

*Hormonal-IUD*: hormonal intrauterine device

### Subcutaneous implant

Subcutaneous implantation of biomaterials-based contraceptives is a combination of progestogen and biomaterials with different structures (e.g., capsules or small rods) that are implanted under the skin and released the drug in vivo steady and constantly. They have a long-acting period and great reversibility. Single implantation can last up to 5 years with a pearl index of 0.1 and full fertility recovery is expected after removal of the implant [41].

#### Non-biodegradable implant

In the 1960s, the Population Council developed the first subcutaneous implanted contraceptive, levonorgestrel silicone rods (Norplant^®^), which consisted of six capsules containing 216 mg of levonorgestrel (LNG) and releasing 40–50 μg of LNG per day [25]. LNG, as a type of progesterone, works on the hypothalamus and pituitary gland, inhibiting the secretion of FSH and LH, which prevents ovulation from being completed, and has significant anti-estrogenic activity [42]. In addition, LNG thickens the cervical mucus and hinders sperm penetration, and can cause the endometrium to thin, which is not conducive to embryo implantation [43]. Although silicone rubber possesses excellent stability, the implant is still considered to be a foreign object in vivo and must be removed when it expires [44]. Moreover, there may be individual differences in the reaction of the human body to silicone rubber, resulting in significant aging of the silicone tubing and difficulty in removal. Jadelle^®^ is a second-generation product, which contains 150 mg of LNG in two silicone rods and is effective for 5 years [45]. Its contraceptive effect is similar to that of Norplant^®^, but the difficulty of removal is greatly reduced by the reduction in the number of implants. Implanon® with ethyl acetate (EVA) used as the carrier matrix is another type of implantable contraceptive extended-release delivery system. EVA not only has good elasticity, flexibility, water resistance, corrosion resistance, but is more tolerant to fillers than silicone rubber. The subcutaneous administration was last for 3 years and the Pearl index was 0.08 [45]. In addition, Nexplanon^®^ is a modified version of Implanon^®^, and the addition of BaSO_4_ allows the use of X-ray-assisted positioning during the operation [46].

#### Biodegradable implant

The biodegradable implant has been studied to avoid secondary trauma to the patients when the device needs to be removed surgically after its expiration. It is delivered through particles loaded with steroids, which are able to ensure both long-term stability with the drug and constant release in biodegradable form. However, both the polymer matrix and the biodegradation products must be innocuous and not accumulate in the body. As a hydrophobic degradable biomaterial, polylactic acid (PLA) is not only capable of controlled degradation but also possesses excellent mechanical and physical properties, tensile resistance, and low biological toxicity PLA forms particles. The releasing system formulated by POE with Na_2_CO_3_ and hydrophobic steroids can prevent liquid in vivo from entering the polymer inside under the prolonged body fluid erosion [47]. Additionally, POE can be hydrolyzed in an acidic environment to release steroids in a time-predictive manner [48]. Meanwhile, it has good biocompatibility as a matrix material, which can be used without causing tissue necrosis or systemic immune reaction. The subcutaneous implant prepared by POE and norethisterone (NET) was pharmacologically studied in baboons and showed that serum levels reached a steady state within 2 days after insertion and remained relatively stable for 5 months [49]. However, local irritation, mild to moderate connective tissue granulomas, and inflammatory reactions were observed in preclinical studies.

As a hydrophobic degradable biomaterial, polylactic acid (PLA) is not only capable of controlled degradation but also possesses excellent mechanical and physical properties, tensile resistance, and low biological toxicity [50]. PLA forms particles with the drug, which are stable in the body, and release the drug at a constant rate. However, degradation of PLA in vivo generates CO_2_ and H_2_O, which can locally produce an acidic environment with a certain probability of causing inflammation [51]. Copolymers prepared from 90% PLA and 10% polyglycolic acid (PGA) incorporate 20% NET to form 90 to 180 μm particles and achieve zero-level kinetic release [52]. Clinical trials demonstrated that 90 days after the operation, the average serum NET levels ranged from 1 to 3 ng/mL. Ovulation was inhibited in all subjects, and NET serum concentrations decreased significantly after 100 days [53].

Compared with biodegradable biomaterials such as POE and PLA which are still in the research and development stage, poly-(ε-caprolactone) (PCL) has been developed for its unique physicochemical properties and its related subcutaneous contraceptive implant is available commercially, called Capronor® [53]. During subcutaneous implantation, the ester bonds of PCL sustain cleavage but maintain the physical integrity until the drug supply is depleted [54]. Further, PCL is degraded to 3000 molecular weight oligomers and finally degraded to carbon dioxide and water [55]. The high permeability of PCL and its copolymers has also been considered as an excellent indicator of drug release diffusion of subcutaneous microcapsules. Feedbacks in clinical phase I evaluation of Capronor^®^ varied significantly between participants, but plasma LNG levels typically remained constant throughout an observation cycle, with average plasma concentrations ranging from 450 to 650 pg/mL [56]. However, cases of interrupted menstrual bleeding patterns occurred in the first 3 months after insertion [57].

### Transdermal patch

The transdermal patch refers to a flake-like preparation made of drugs and biomaterials that can be adhered to the skin and produce systemic or local effects [58]. The drug can be delivered to the skin through the surface layer of human skin non-invasively [58]. Compared to oral administration or subcutaneous implantation, transdermal patches can avoid incompatibility of the drug with the gastrointestinal tract or first-pass metabolism. In addition, the sustained drug release in the patch maintains a stable effective blood concentration with reduced side effects associated with burst release. Besides, the frequency of patchoperation is greatly reduced compared to oral and injectable administration, as well as being self-administered by the patient, which greatly improves adaptability and acceptability. Furthermore, the characteristics of the material itself are also crucial for patch application, e.g., homogeneity and release with the drug, adhesion and permeability to the skin, microbiological limits and the degree of drug residue.

Ortho Evra® is the first transdermal contraceptive patch to be marketed, and it was approved by the U.S. Food and Drug Administration (FDA) in 2001 [59]. The patch consists of three layers. The backing layer is composed of a beige flexible film made up of a low-density pigmented polyethylene outer layer and a polyester inner layer. It provides structural support and protection. The middle layer contains polyisobutylene/polybutene adhesive, crospovidone, non-woven polyester fabric, and lauryl lactate as inactive components. The active components in this layer include 0.75 mg of ethinyl estradiol and 6 mg of methyl progesterone, which release ~ 35 μg of ethinylestradiol and 150 μg of norgestimate per day. The third layer is the release liner, which protects the adhesive layer during storage and is removed just before the application [59]. The patch is used continuously for 1 week, and its compliance is greatly improved compared to the oral contraceptive once a day. The effect of avoiding pregnancy is satisfactory, and the Perl index in clinical experiments is only 1.07 [60].

#### Dissolving microneedle (DMN)

**Fig. 4:**
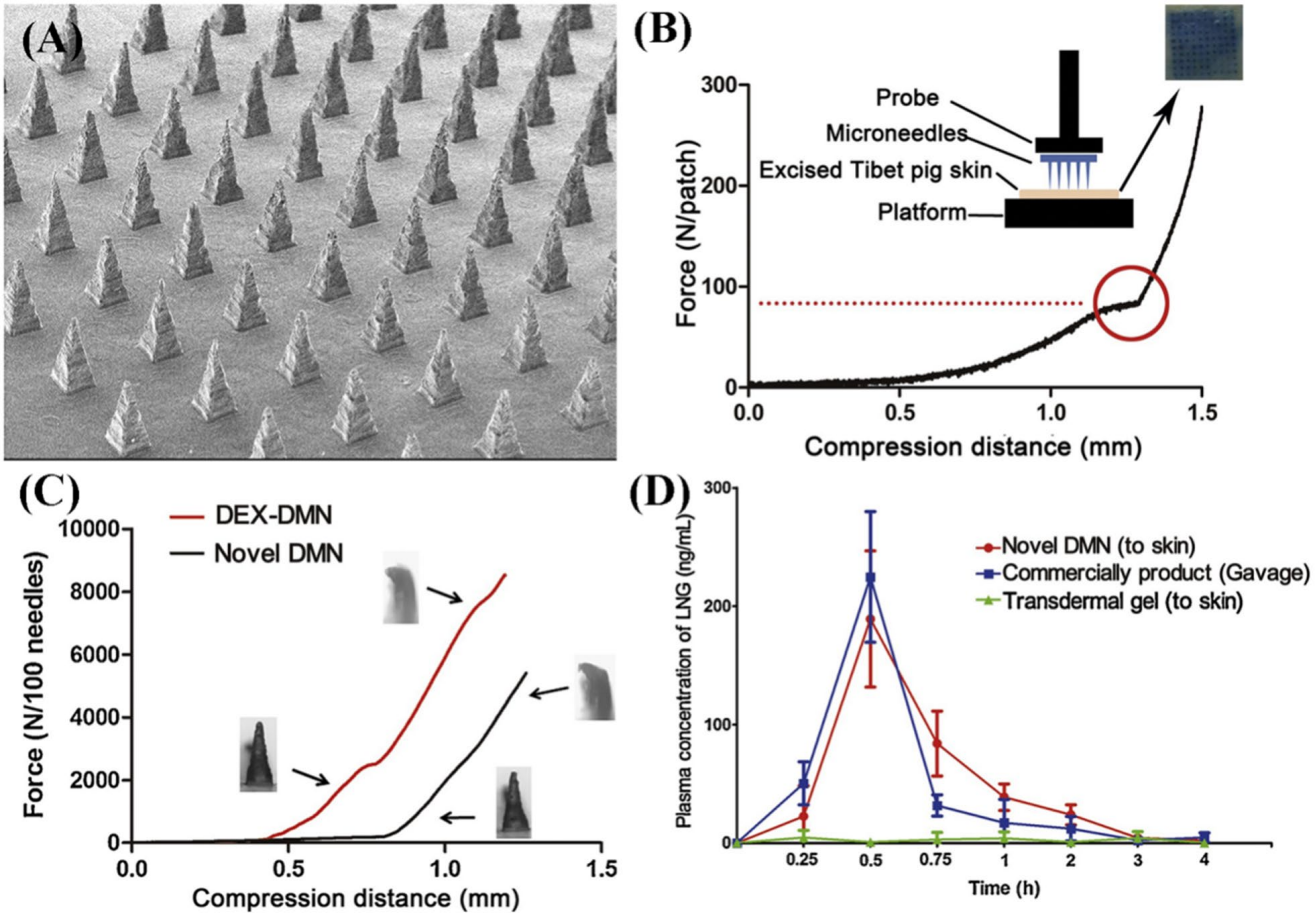
**A** Scanning electron microscopy (SEM) image of DMNs (LNG-HP-β-CD). **B** Skin penetration test of DMNs. **C** Detection of mechanical properties of DMNs with or without chitosan and β-GP. **D** Pharmacokinetic assay of LNG loaded into various groups (Reprinted with permission from reference [30]. Copyright 2017 Elsevier, Ltd)

**Fig. 5:**
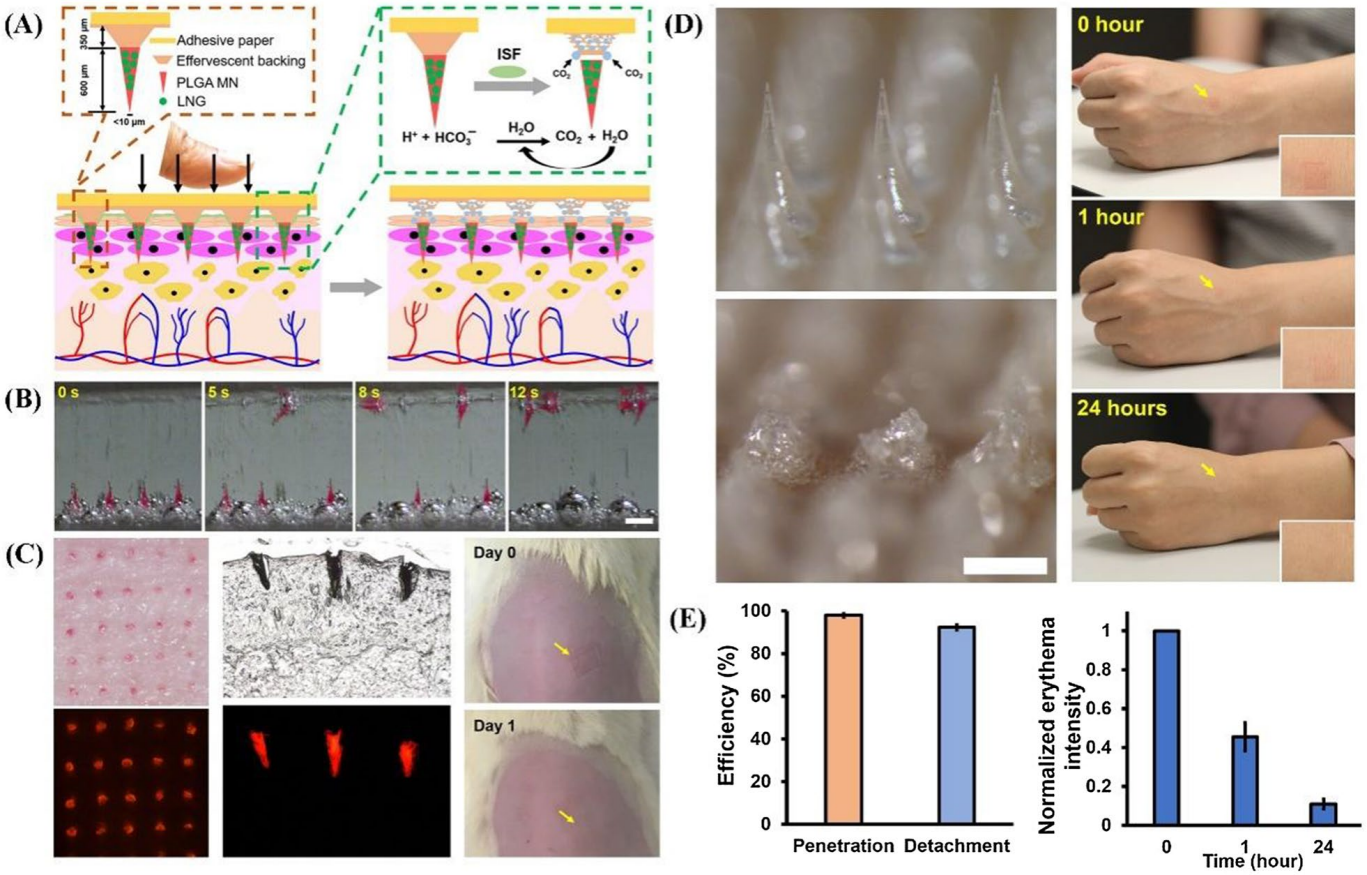
**A** Schematic representation of the design of the MNs with bubbly sponsorship and the interaction of application to fast convey MNs into the skin. **B** Rapid detachment of MNs patches from effervescent backing. **C** Representative images of skin, tissue section, and rat after MNs application and removal. **D** Structural changes of MNs before and after application and human skin reaction in 0–24 h. **E** The efficiency of penetration and detachment of the microneedles and Normalized erythema intensity of human skin in 0–24 h (Reprinted with permission from reference [8]. Copyright 2019 American Association for the Advancement of Science)

Recently, microneedle has been developed as a type of micron-sized drug delivery channel into the skin, allowing large molecules to pass through and only puncture the stratum corneum without touching the nerve endings, so there is slight pain or even no pain [61, 62]. DMN is composed of biocompatible and biodegradable polymers that degrade in the body, dissolving in the skin at a constant rate and releasing the encapsulated drug gradually, leaving no acrid or harmful biological waste [30]. However, there are still some hardships to be solved that is how to promote the dissolving ability and drug loading proportion. The poor dissolving ability of the carrier always leads to inefficient drug release and potential skin irritation. Thermosensitive materials containing CS and β-GP can improve the dissolving ability of DMN by temperature-induced phase transition [30]. In addition, hydroxypropyl beta-cyclodextrin (HP-β-CD) enhances the solubility of low water-soluble steroids by forming an encapsulated compound, which increases significantly the limited drug loading [63]. Yao et al*.* presented a novel type of DMNs with great mechanical strength, dissolving ability, and drug release profile in vitro [30]*.* LNG was encapsulated into HP-β-CD to form inclusion compounds, while the DMN was prepared with the inclusion compounds and thermosensitive materials containing chitosan and β-GP. The DMN has successfully penetrated the skin without skin irritation, and the DMN was dissolved 69.32 ± 4.23% within 2 h, which was almost twice the dissolution rate of conventional DMN. In addition, HP-β-CD loaded with LNG increased the drug loading capacity to 3380.56 μg/mL, exhibiting a similar pharmacokinetic curvature as oral LNG suspensions (Fig. 4). PLA and polylactic acid co-glycolic acid (PLGA) were chosen as microneedle materials since these biodegradable polymers offered the advantages of biocompatibility and mechanical properties, with LNG loading over 40% and sustained slow release for weeks to 11 months [64, 65]. Li et al*.* reported a microneedle patch design with degradable PLA and PLGA on the application of sustained-release LNG. (Fig. 5) [8]. The separated microneedles were then slowly biodegraded in the skin to control the constant release and systematic delivery of encapsulated LNG for more than a month. In the rats model, microneedle patches were well tolerated with few signs of use and maintained plasma hormone concentrations above human therapeutic levels within a month.

To address the rapid separation between the microneedle and patch backing, Li and co-workers developed an effervescent backing composed of sodium bicarbonate and citric acid, which can react to the interstitial skin fluid from solid to the gas state, achieving effective separation of the microneedle in less than 1 min (Fig. 5) [8]. Simple and rapid separation can greatly reduce the patient’s degree of sense of pain during detachment, the risk of skin damage, and the loss of active microneedle components. Similar phenomena were also observed in the bubble structure and porous structure between the microneedle and the backing [66, 67]. Li et al*.* reported that the bubble substrate formed by water-soluble polyvinyl alcohol and sucrose has strong compression resistance but weak shear resistance without changing the size and shape of the microneedle, which can achieve rapid separation after being inserted into the skin [68]. In addition, Lee et al*.* proposed that the porous structure of the platform pad was designed to provide sufficient pressure support for complete insertion into the skin and rapid separation under shear force during detachment in less than 1 s [69]. Polyvinylpyrrolidone (PVP) is considered to be an ideal material for the preparation of porous structures, which can be optimized for porosity and mechanical properties by adjusting the PVP/H_2_O ratio [70]. As a matrix carrier of the microneedle, PVP with great biocompatibility and high mechanical stability would not cause shrinkage or fracture during the freeze-drying process.

### Oral administration

**Fig. 6:**
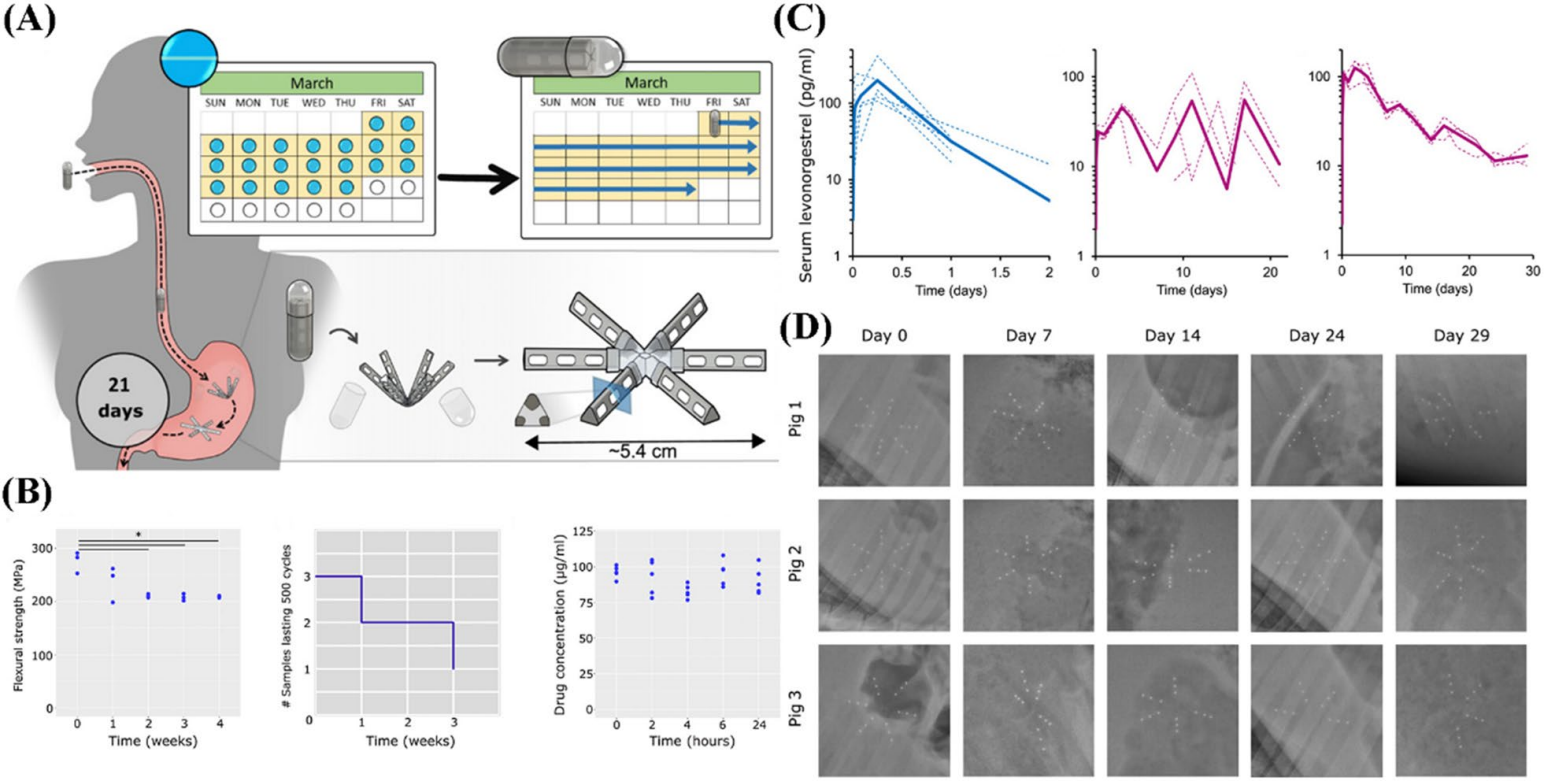
**A** The design of the PDMS-based polymer matrices for oral drug delivery. **B** The performance evaluation of the arm includes flexural strength, interfacial tensile stability, and stability of LNG in the devices. **C** Detection of serum concentration of LNG in pigs administered as pills (left), three arms of PDMS (middle), and six arms of PDMS (right). **D** X-ray images observing the arm shapes and retention sites in the porcine stomach (Reprinted with permission from reference [10]. Copyright 2019 American Association for the Advancement of Science)

Traditional oral contraception needs to be administered for 21 days continuously during a menstrual cycle, which greatly increases the risk of missed medication. The missed medication causes lower progesterone levels in the body, abnormal follicle development, and thin cervical mucus. The rate of unplanned pregnancies due to missed doses is as high as 9% with an increased risk of abnormal fetal growth [71]. Biomaterial-based oral contraception is expected to be self-administration with a lower dosing frequency for up to 1 month. And it will have a lower risk of damage and infection to the gastrointestinal tract due to the superiority of the structural design and antimicrobial properties of biomaterials. For instance, Kirtane et al*.* proposed that an orally administered long-acting gastric resident dosage form composed of V-shaped six polymer arms loaded LNG and connected by an elastomer core, which allowed it to be folded into a capsule for easy oral administration [10]. After the capsule dissolved in the stomach, the device would form recoils in a larger size than that of the pylorus, so that it could be fixed there and release the drug constantly and steadily (Fig. 6). Importantly, the form consists of Polydimethylsiloxane (PDMS) and poly (sebacic anhydride) to carry LNG for long-term drug release. As an inert polymeric biomaterial, PDMS with good plasticity biocompatibility, biostability, and effective control of drug release has been widely used in a series of drug delivery devices [72–75]. Poly (anhydride) features not only a larger total drug release but also the release rate that can be adjusted by controlling the monomers used to synthesize the polymer [76]. Pharmacokinetic studies showed that comparable concentrations of LNG were produced on days 3, 11, and 17, revealing a unidirectional variability in serum concentrations. In addition, animal tests showed that 16 of 18 arms remained in the pylorus after 30 days without any detachment. This form is expected to address low drug adherence compared to oral pills and achieve effective contraception for up to 1 month.

### Vaginal contraception

The vagina has the unique anatomical advantage of serving as the only route for sperm to enter the woman’s body during intercourse, while the elastic folds of the vagina itself provide the basis for long-term device placement. Compared to the gastrointestinal tract, vaginal long-acting contraception has lower drug interactions and doses of drugs, greatly increasing the biocompatibility and bioavailability of the device. Faster hormone absorption in vaginal epithelium, sustained local release with a lower risk of triggering hormonal side effects made the ring more appealing. In addition, long-acting vaginal contraception can significantly improve user compliance compared to subcutaneous implants, thus ensuring better contraceptive outcomes.

**Fig. 7:**
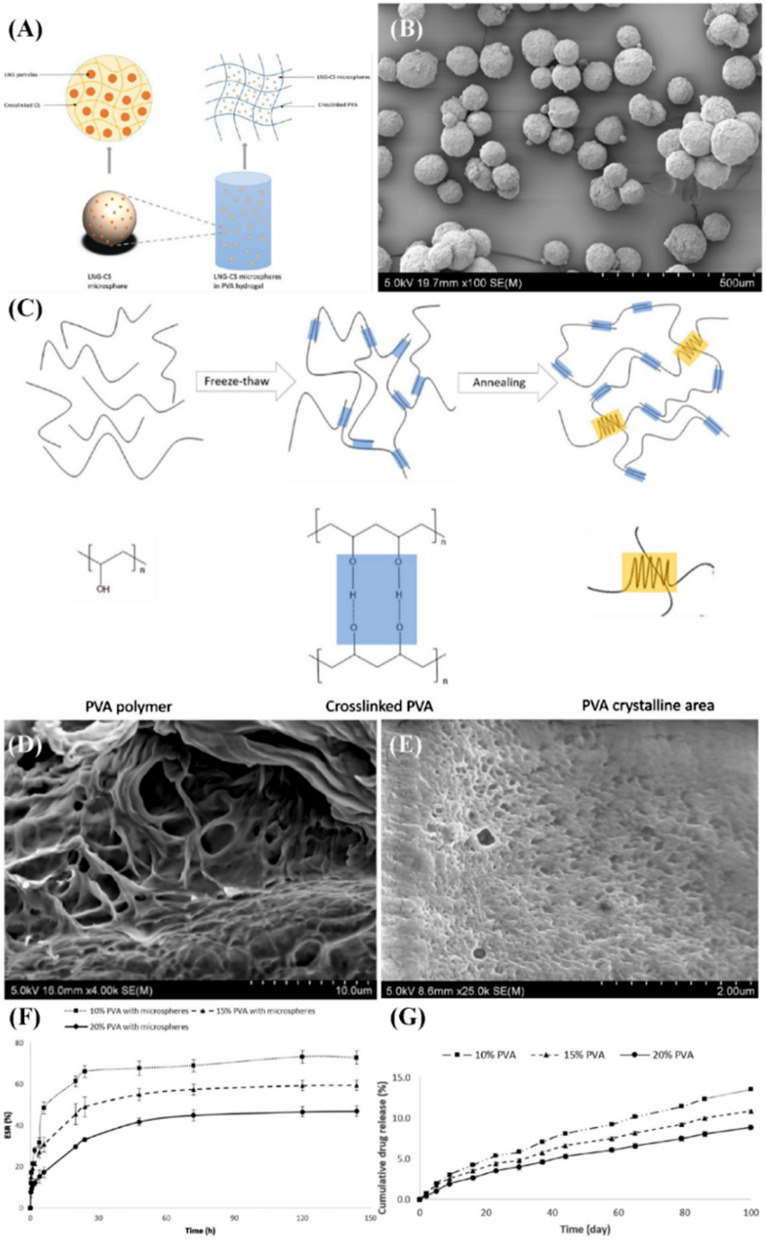
**A** Schematic representation of the fabrication of CS-LNG microsphere in PVA hydrogel. **B** SEM micrograph of CS-LNG microsphere. **C** Schematic diagram of PVA hydrogel by hydrogen bonding and annealing. **D** SEM exhibition of 10% PVA hydrogels after drying and annealing. **E** Dissolution test observed by SEM micrograph of 10% PVA hydrogel combined with LNG-loaded chitosan microsphere. **F** Equilibrium swelling ratio of PVA hydrogels. **G** Cumulative LNG release from PVA hydrogel in vitro (Reprinted with permission from reference [9]. Copyright 2019 American Chemical Society)

The vaginal ring is an intravaginal cyclic contraceptive device made of hormones together with polymeric compounds with slow release capability [77]. The hormone can be absorbed through the vaginal epithelium into the local or systemic blood circulation, inhibiting follicle development and ovulation, which is safe, reversible without the first-pass effect [78]. It can be placed and removed by the user itself, which greatly improves compliance, but it has the potential to irritate the vagina. Silicone rubber polymer was earlier used as vaginal contraceptive carriers because of the inert silicone elastomer and the larger surface ratio, which allows the carrier to bind more drugs and hormones such as medroxyprogesterone acetate (MPA), LNG, and E2, increasing the duration period of function [79, 80]. Nevertheless, clinical trials showed that silastic rings always presented a high incidence of nausea and vomiting, particularly in the first cycle of use, which attributed to the accumulation of drugs on the surfaces of rings causing the initial burst effect [79, 81]. To address the issue above, polyethylene vinyl acetate copolymers might also update silastic polymer to show into the following technology. Laarhoven et al*.* presented a vaginal ring consisting of ethylene–vinyl acetate copolymer which exhibited suitable permeability properties that could make a controlled release system with specified release characteristics, values of the diffusion coefficient, and solubility [82]. In this polymer system, it is feasible to load steroids (e.g., ethinylestradiol and ethinylestradiol) to make certain of the effectiveness of the birth control and to reduce the negative effects of each steroid [83]. The application of biodegradable polymers like PLA overcome the responding blockage since the release is controlled by the degradation of the polymer instead of the solubility and subsequent diffusion of the drug through the polymer [84]. Conville et al*.* demonstrated a hydrogel delivery system consisting of a blend of polyethylene vinyl acetate (PEVA) and polylactic acid with hydrophilic tenofovir, which exhibited a desirable performance in a long-acting slow-release by adjusting the ratio of PLA to PEVA for contraception, pregnancy, and blocking HIV transition [85]. However, hydrogel rings can also present some issues which are always difficult to regulate, such as poor vaginal drug loading and abnormal vaginal discharge [86]. Long et al*.* proposed a controlled releasing system in which LNG was chemically cross-linked and encapsulated in CS to form CS-LNG microspheres [9]. The microspheres had a concentrated particle size distribution and significantly prolonged the drug release. Besides, the drug release can be more extended and controlled through incorporation into PVA hydrogel with different concentrations (Fig. 7). Saxena et al*.* fabricated non-biodegradable 2-hydroxyethyl methacrylate and sodium methacrylate, as well as soluble acacia gum hydrogel rings for delivery of anti-HIV agents and non-hormonal contraceptives [87]. The prepared rings showed sustained release for 10 and 28 days in vitro. Nonetheless, a potential risk associated with hydrogel ring is the tendency to absorb aqueous fluids and swell, which can doubtlessly reduce the ability to release drugs and the range of drug interactions with the body in vivo [88]. Sharifzadeh et al*.* presented a montmorillonite (MMT)-based polyacrylamide hydrogel ring for controlled vaginal drug delivery [89]. MMT sustains drug release via strongly adsorbing to the drug molecule, which improves the bioavailability and dissolution rate of hydrophobic drugs and even more great swelling within controlled in vitro release.

### Intrauterine device (IUD)

IUD is a long-acting contraceptive device placed in the uterine cavity, which is classified into copper/hormonal-IUD. It has advantages over other methods in terms of great contraceptive effectiveness, easy administration, low cost, and spontaneous reversibility [90]. The device exerts contraception effect through interfering with fertilized egg implantation, causing a non-inflammatory endometrial response, and preventing binding between sperm and egg. Recently, significant progress has been made in the development of IUDs, with some well-established products like Cu-IUD, hormonal-IUD, frameless IUDs, and unique insertion devices being commercialized (Table 4**)**. However, adverse effects such as uterine bleeding in pregnancy with the device and abnormal vaginal discharge after IUD insertion during IUD use have also been caused widespread concern [91].

**Table 4:** Commercial products of IUD [92–94]

Product	Channel	Characteristics	Pearl index
CuT380A	Copper ion Cu^2+^ release	① Nonhormonal, and effective emergency contraceptives ② Increased incidence of bleeding and cramping	0–0.8
Viracept	Cu^2+^ release	① Lower total copper loading ② Great performance on flexibility	0–0.45
IUB	Cu^2+^ release	① Shape memorized alloy ② Potential risk of expulsions and removals	0–0.29
Mirena Liletta Skyla	Hormonal delivery	① *Skyla* with the lower LNG reduces pain bleeding	0–0.14
GyneFix 200	Frameless IUD	The flexible shape reduces pain and ectopic pregnancy	1.3

*IUB*: the intrauterine ball

#### Copper-IUD (Cu-IUD)

The mechanism of Cu-IUD is considered that the release of Cu^2+^ causes the inactivation of sperm and the suppression of myometrial contractions [95]. Cu is gradually oxidized and corroded to release Cu^2+^ after exposure to the uterine cavity [96]. Yet in practice, although the CuT380A is superior to other devices in contraceptive efficacy, bleeding and pain due to drainage and removal were still emphasized [97]. The crucial reason lies in the unstable release rate of Cu^2+^ and by-products generated in the corrosion process. Direct contact with the uterine fluid causes the rapid oxidation and corrosion of the Cu on the surface of the device. During the initial implantation, Cu^2+^ is released in amounts of up to 296 μg/day, known as the initial burst release of Cu^2+^ [98]. When the concentration of Cu^2+^ exceeds 80 μM in the uterine, it causes spasmodic muscle contractions, which in turn trigger irregular vaginal bleeding, evacuation, and lumbosacral pain [99]. It is necessary for Cu-IUD to ensure a prolonged and effective Cu^2+^ release concentration. The corrosion of Cu also produces the by-products such as Cu_2_O, Cu(OH, Cl)_2_·2(H_2_O), and Cu_2_(CO_3_)(OH)_2_, which can adhere to the Cu-IUD surface and prevent the release of Cu^2+^ [100, 101]. The deposits can adhere to the Cu-IUD surface and prevent the release of Cu^2+^, negatively interfering with the effect of birth control. Therefore, it is imperative to balance the initial burst release of Cu^2+^ at an early stage with the effective contraceptive concentration over a long period.

A nano-Cu/low-density polyethylene (Nano-Cu/LDPE) as a copper carrier in IUD was reported by Hu et al. [102]*.* Copper nanoparticles were combined with LDPE by physicochemical methods to form a composite allowing to the nanoparticles be uniformly distributed in the composite material. Its internal spacing provides penetration channels for Cu^2+^ and corrosion media, and the corrosion rate is effectively controlled by separating the copper nanoparticles from the LDPE, enabling a rapid and constant release rate within 5 h [103]. Besides, tissue toxicity has not been observed in nano-Cu/LDPE during the acute and sub-chronic treatments [97]. It has increased contraceptive effectiveness up to 5 years, which is suitable for the long-acting reversible contraceptive channel [104]. A novel type of alloy made of magnesium (Mg) and ultra-fine grained bulk Cu (UFG-Cu) was also reported to tailor the burst release of Cu^2+^ in Cu-IUD [105]. Mg exhibits good biocompatibility and degradability and as an essential element of the human body, Mg^2+^ participate in metabolism and regulate cell proliferation and apoptosis. The addition of Mg to UFG-Cu allows dissolving preferentially compared to Cu, effectively avoiding the explosive release of Cu^2+^, which can be kept constant during long-term use to ensure effective contraception [105]. In addition, the incorporation of Mg can improve the histocompatibility of Cu-IUD in the uterus with a significant reduction in the side effects of the device. The copolymer (CuCl_2_/SiO_2_/PVA) was also synthesized by putting Cu in the form of Cu^2+^ to overcome the inherent defects of the Cu corrosion process [106]. Polyvinyl alcohol (PVA) is a non-toxic polymer material with great biocompatibility and exhibits minimal cell adhesion and protein absorption ability [107]. Significant improvement in strength, permeability, and biocompatibility after modification by silica sol [108]. In the composite, the chelation equilibrium determines the release of Cu^2+^, avoiding the formation of oxide by-products and improving the effective utilization of the material [108]. Other biopolymer composites such as chitosan/alginate multilayer film and PDMS-based electropolymeric coating also play effective impacts on controlling the burst-releasing and stability of long-term contraceptive effect, as detailed in Table 5.

**Table 5:** The development of solving the “burst-releasing” phenomenon of Cu^2+^

Materials	Bio-component	Technological breakthrough	Ref.
IDM delivery system	Indomethacin	① The initial explosion release of Cu^2+^ was eliminated ② The sustained release time of IDM was controlled by adjusting the number of layers of the film	[109]
Novel Cu/PDMS nanocomposite	Nano-Cu particles	① The distribution of Cu nanoparticles was more uniform in the PDMS matrix ② The release rate of Cu^2+^ became adjustable by changing the number of Cu nanoparticles	[110]
*CuT380* IUD with PLGA film	PLGA film	The occurrence of the burst release of Cu^2+^ was relieved by coating PLGA on the *CuT380*	[111]
Phytocompound-based electropolymeric coating	PolyCarvCu coating	It was a strong inhibitory effect on the initial release of Cu^2+^	[112]

*IDM*: indomethacin

#### Hormonal-IUD

Hormonal-IUD is used to prevent pregnancy by containing hormones that interfere with the normal menstrual cycle, and the long-term release of hormones in the uterus suppresses ovulation and changes the properties of the cervical mucus, which is unfavorable to prevent the entry of sperm into the uterus and fertilization. However, the unstable release of hormones has always been a concern. PE has been regarded as a drug delivery matrix for hormonal IUD, which is not only because of its reasonable surface area and release rate after carrying hydrophobic hormones, but also its stiffness and hardness for use in the design of IUDs [113]. PE-progesterone matrix used for IUD as a-helices was be designed by Kalkwarf et al. [26]. However, the progesterone release rate was as high as 225 μg/day/cm^2^ in the early stage and was relatively constant at 14 μg/day/cm^2^ until the 6th day [26]. The release rate of 30 μg/day/cm^2^ in the uterus would prevent contraception without affecting normal ovulation [114]. Therefore, the controlled release of hormones has become a problem to be solved for hormonal-IUD. Nilsson et al*.* put forward a Norgestrel-PLA film fixed on T-IUD [114]. PLA film is suitable to prepare the long-acting controlled IUD due to the great diffusion and hydrolysis. Clinical trials showed that circulating ovarian function could maintain during intrauterine injection of norgestrel [114]. But the sharp reduction of menstrual blood during the menstrual cycle is one of the main causes it has not been widely used.

**Fig. 8:**
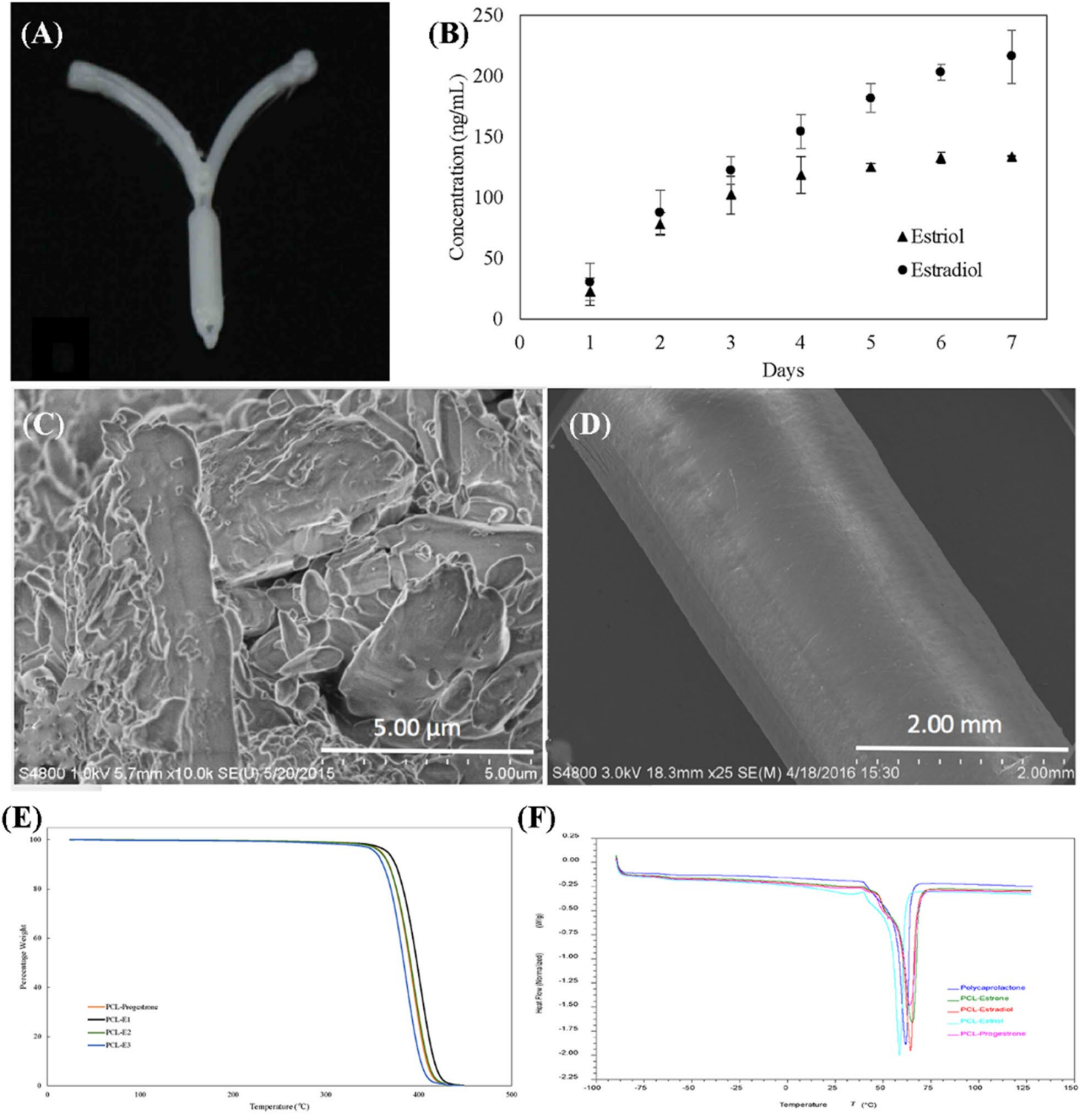
**A** Macrostructure of 3D-IUD. **B** Cumulative release assay of estriol and estradiol from 3D-IUD. **C**, **D** SEM micrograph of surface morphology of coated pellet and filament. **E**, **F** Thermogravimetric analysis and differential scanning calorimetry of PCL-hormones composites (Reprinted with permission from reference [115]. Copyright 2017 Public Library of Science)

The great potential will be explored in the field of personalized contraception through 3D-printing engineering. Factors such as material structure, hormone dosage, and hormone treatment cycles are directly considered in the design to achieve contraceptive purposes better and reduce side effects significantly. A customizable and biodegradable 3D-printed IUD was proposed by Tappa et al. (Fig. 8) [115]. The estrogen and progesterone are encapsulated in PCL, and the intrauterine contraceptive device with a shape that can be adapted to the body's structure is prepared by fused deposition modeling. As a linearly synthesized degradable aliphatic polyester, PCL possesses high mechanical strength, plasticity, biocompatibility, and low degradation rate, while its degradation products are completely metabolized by the tricarboxylic acid cycle or directly excreted by the kidneys, which makes it suitable for long-term carrier implantation in vivo [28, 116]. In addition, PCL has been approved by the U.S. FDA for drug delivery system applications with a good drug delivery structure and stable drug release rate [9]. The 3D-printed IUD showed an extended hormone release over 7 days in vitro [115]. But the steroid concentration of the implants is merely 1% w/w, therefore it will be a meaningful research direction to improve the drug loading capability of 3D-printed IUDs through the superior performance and structural design of biomaterials.

### Fallopian tube occlusion

The traditional route of contraception through the fallopian tubes focuses on occluding or removing the tubes permanently, thus preventing the egg from binding to the sperm and the fertilization. The main approach is to inject corrosive drugs into the fallopian tubes, destroying the mucous membrane, permanent scarring, and eventual occlusion of the tube lumen for permanent contraception. However, there is no doubt that side effects, including severe pain, inflammation, tubal perforation, abnormal menstrual cycles, pelvic adhesion, and some reproductive cancer risks, cannot be unavoidable [117, 118]. And there is increasing attention on the use of biomaterials for female tubal occlusion due to its low interference with the female endocrine system, low chance of pelvic infection, and simple and effective operation.

#### Permanent tubal occlusion

For permanent tubal occlusion, the ideal device must equip properties with high efficiency, great mechanical performance, fabricability, and tissue reactivity [119]. Established tubal occlusion materials, such as silicone rubber, PET, methanoacrylate, and situ-gelling materials formed by Michael-type additive reaction, have been marketed [120]. However, in recent years, researchers have been working on changing the physical state of such materials and combining them with other substances in order to achieve the high contraceptive effect with significantly reduced side effects. Guo et al. proposed that polidocanol were regarded as a detergent-based hardener in the form of foams rather than liquids, which can improve the contact area of the material with the epithelium, increase foam stability while reducing drug concentrations and potentially toxic epithelium-damaging activity [121]. The addition of benzalkonium chloride (BZK) has been shown to improve the stability of PF. Abdala et al*.* demonstrated a cross-linked type I bovine collagen stent that rapidly absorbed fluid and maintained structural elasticity with uniform pore size, structure, aspect ratio, and distribution for permanent tubal occlusion [122]. Obvious tubal obstruction and peripheral vascularization with degradation of the stent material and luminal filling with immune cells were observed after 12 days [122]. The results showed low cell density and significant collagen deposition in the structure.

#### Reversible tubal occlusion

Permanent sterilization may cause severe tubal damage and reduced fertility. Therefore, an increasing number of couples are seeking reversible, painless, and safe tubal contraceptive methods. In contrast to silicone rubber alone, Wang et al*.* proposed a nickel–titanium shape-memory alloy wire as a tubal plug for reversible contraception [123]. The framework consists of silicone rubber and nickel–titanium shape-memory alloy filaments with a cylindrical center and a petal-like Amphi-terminal. The nickel–titanium alloy not only has good biocompatibility, wear resistance, and corrosion resistance, but also allows a combination of perception and shape reversibility [124, 125]. Only 1 of 30 rabbits was pregnant after insertion of the plug, and all animals in the previous contraceptive experiments were pregnant after removal of the plug [123, 126, 127].

**Fig. 9:**
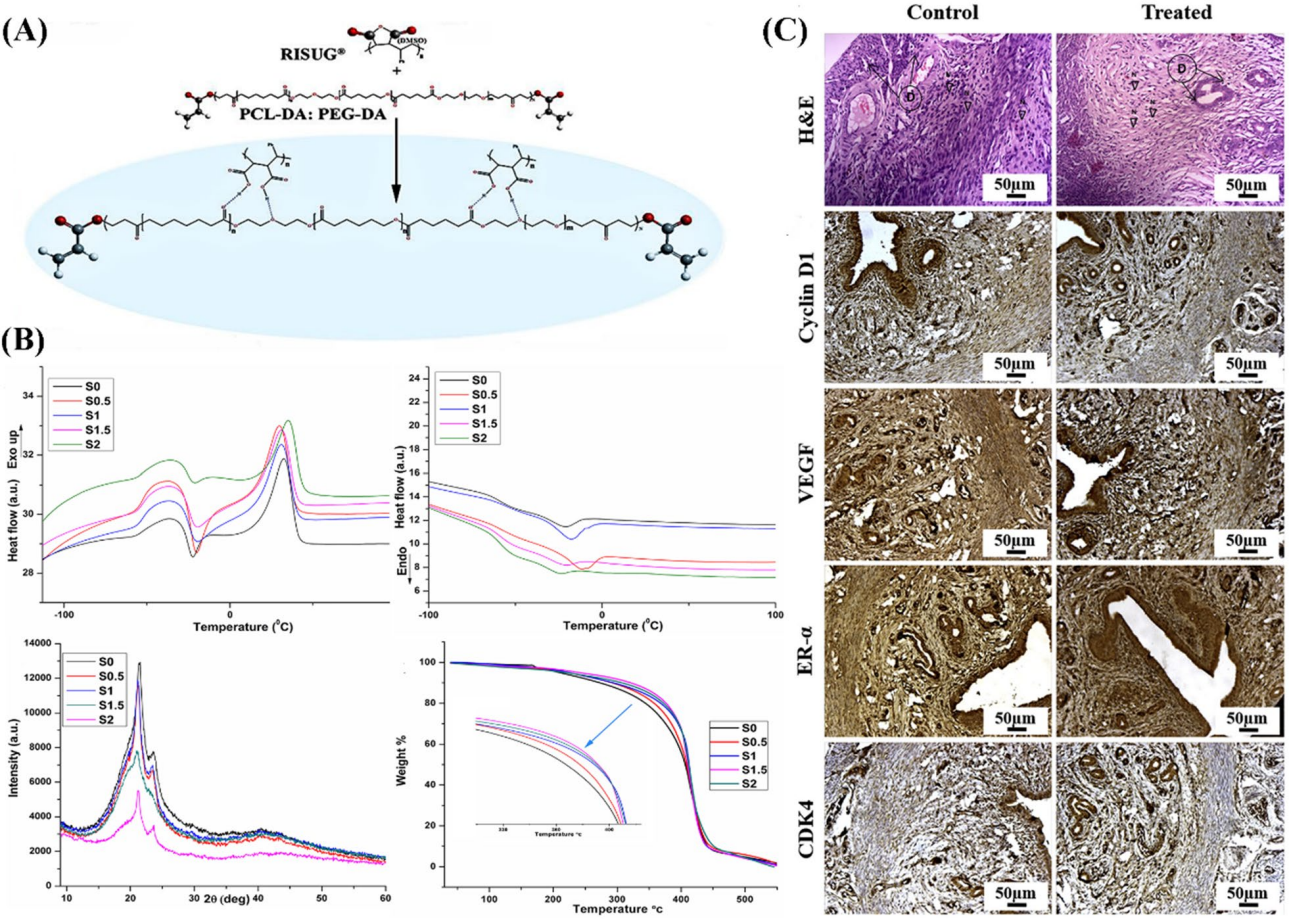
**A** Schematic diagram of semi-interpenetrating polymer network composed of PCL-DA, PEG-DA, and RISUG^®^. **B** The characterizations of polymer containing cooling and heating curves, X-ray diffraction peak patterns, and thermogravimetric analysis. **C** Immunohistochemical evaluation of rat uterine tissues compared with control and treated groups (Reprinted with permission from reference [129]. Copyright 2020 Elsevier, Ltd)

In addition to the metallic materials, researchers have attempted to utilize biodegradable polymeric materials as candidates for tubal contraception because of their controlled reversibility. Abdala et al*.* used ethylene–vinyl alcohol (EVOH) copolymers to plug one of the fallopian tubes in a rabbit model [122]. EVOH copolymers are biocompatible polymers consisting of a random mixture of hydrophobic ethylene and hydrophilic vinyl alcohol. DMSO has diffused away from the mixture when the mixture comes in contact with aqueous media, causing the polymer to settle and solidify in situ and forming a spongy plug that seals the lumen [128]. The copolymer was successfully transferred into the oviducts of all rabbits and no fertilized egg injection sites were observed. However, the only one case was the majority of the embolic agent immediately extruded from the oviduct into the uterus, and histological analysis showed varying degrees of occlusion, fibrosis, and inflammation. Subramanian et al. represented an innovative insight into the use of a hydrogel formulation composed of styrene-cis-butylene-based styrene maleic anhydride (SMA) dissolved in dimethyl sulfoxide (DMSO) to make a non-hormonal tubal contraceptive implant [129]. Polymeric hydrogels cause sperm inactivation by lowering the pH of the surrounding environment to disrupt the acrosome. The low pH not only destroys the acrosome but also alters the morphology of the sperm, leading to low sperm viability and consequently the loss of sperm fertilization. This compound has been widely used for vas deferens injection of reversible male contraceptives and clinical trials have shown a positive trend in birth control [130, 131]. Meanwhile, the hydrogel implanted in the isthmus of the uterus did not show any implantation swelling, dilation, arrest, contraction, or signs of abnormal growth on the outer surface compared to the control group (Fig. 9) [129].

### Vas deferens contraception

Among the traditional male contraceptive methods, condoms, hormonal contraception, and vasectomy have become well-established and effective. However, the estrogenic side effects of steroids and the irreversibility of post-vasectomy have prompted the novel male contraceptive method with characterizations embodying one-time intervention, lengthy-appearing results, low side outcomes, and alternatives of reversibility [132]. Vas occlusion allows for a single intervention, easy administration, good contraceptive effectiveness, and reversible fertility. And the method has almost no side effects on the body and little impact on the sexual experience. More importantly, the choice and design of biomaterials enable the approach to avoid the use of hormones. Light and heat stimulation are alternative surgical options to achieve non-invasive fertility restoration.

#### Reversible filtering-type intra-vas device (IVD)

In comparison to vasectomy, IVD has both good contraceptive efficiency and the ability to maintain the patency of the vas, which greatly reduces the incidence of complications [133]. Various types of IVD have been developed, such as threads, copper wires, electrical devices, etc. [134–136]. Among them, the Cu-IVD shows a good spermicidal effect and antibacterial performance [137]. However, the burst release of copper ions and the formation of CuO on the surface of IVD have been observed, which greatly increases the biological toxicity and limits the long-term use of Cu-IVD. Cross-linked composites containing polyvinyl alcohol (PVA), nanoparticles of silica, and CuCl prepared for IVD can also substantially improve outburst release and metallic copper oxidation. Chen et al*.* reported that a novel filtering-type IVD was made of nano-SiO_2_-Cu^2+^ complex crosslinking polymer composites [133]. Plenty of micropores in the wall of the IVD prototype can be employed to filter the sperm while keeping the vas deferens unobstructed. Besides, the burst release of Cu^2+^ could be avoided in IVD. The effective utilization of Cu^2+^ can be achieved in this novel copper-containing composite and it did not cause obvious toxicity for the cells of the male reproductive organs after 1 year of implantation [138].

#### Reversible vas occlusion

**Fig. 10:**
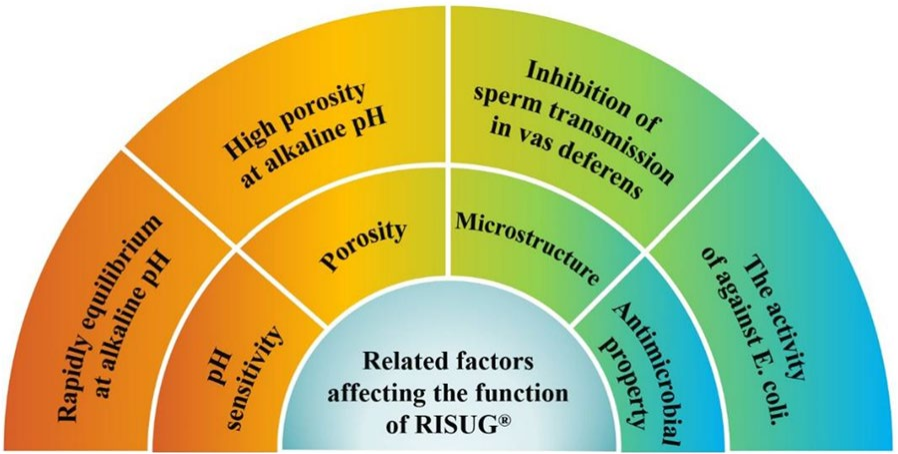
Related factors affecting the function of RISUG [144, 145]

RISUG® has been reported as a category of male contraception with reversible vas occlusion. SMA copolymer is injected together into the vas deferens, which reduces the pH around the microenvironment and creates a positive charge leading to sperm inactivation [107, 110]. The flakey precipitate is formed in the lumen through injecting RISUG® just like a labyrinth channel when sperm passed brushing under SEM. Additionally, the sperm membrane can be subjected to an electric charge and pH stress brought by the precipitate, impairing acrosome function and causing the leakage of enzymes necessary for fertilization [109, 110]. NaHCO_3_ and DMSO are both able to dissolve SMA at alkaline pH conditions, which provides a relatively reversible tendency through injecting NaHCO_3_ and DMSO into vas deferens [139]. Theoretically, DMSO exhibits a highly reactive sulfur portion, which interacts with the ether oxygen (–O–) of SMA to form SMA-DMSO complexes and dimethyl maleic anhydride [130]. Related properties and influences of RISUG® had also been reported in succession (Fig. 10). Measurements in rats showed 100% contraceptive efficiency after 90 days of RISUG® injection and achieved satisfactory reversible fertility after 30 days of NaHCO_3_ injection [140, 141]. Similar phenomena were also observed in VasalGel™ [142]. The high molecular weight (360 kDa) SMA polymer acts as a physical-charge barrier to block sperm passage. It has been tested in rabbits and rhesus monkeys with satisfactory results in contraception and fertility recovery [143].

**Fig. 11:**
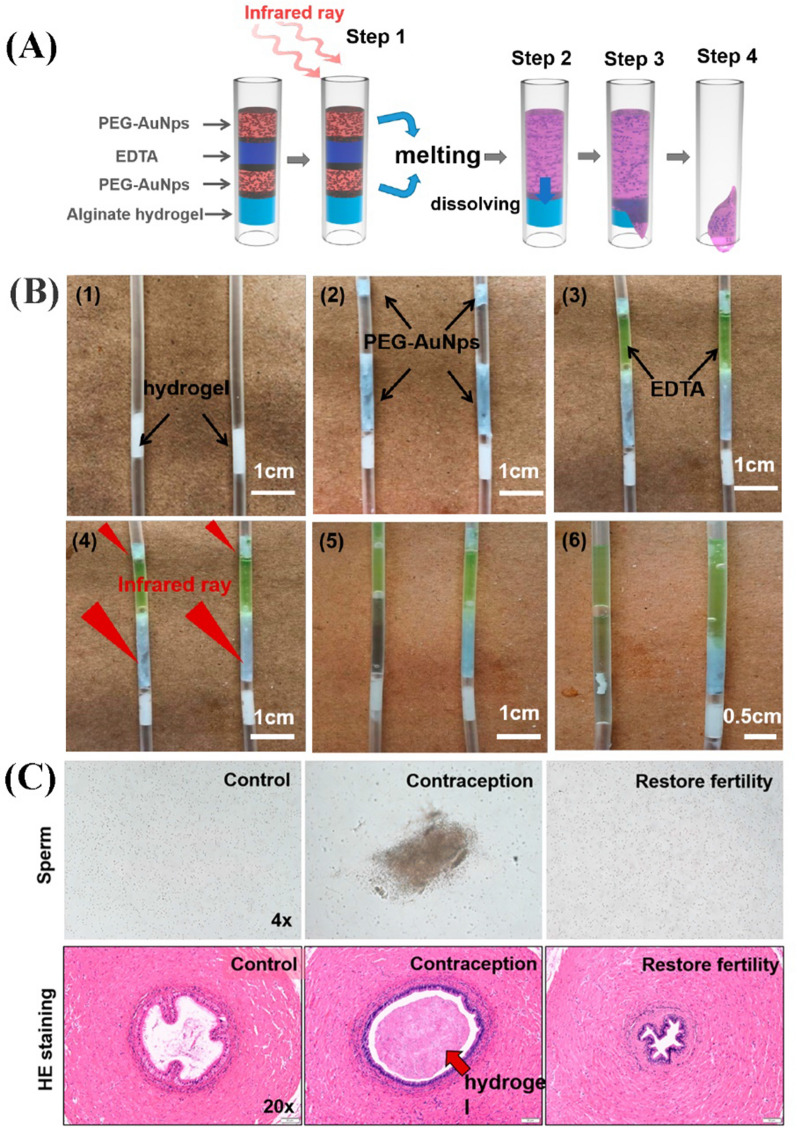
The medium-term male contraceptive composite hydrogel system via physical and chemical inhibition. **A** Composition of the four components of the system in the tube and the dissolving process after Infrared ray irradiation. **B** in vitro stimulation of fertility recovery after Infrared ray irradiation. **C** sperm morphology analysis from vas deferens and HE staining from the cross-section of each the vasculature of three types of rats (Reprinted with permission from reference [29]. Copyright 2019 American Chemical Society)

Recently, a new long-lasting contraceptive composite hydrogel system has been able to achieve both physical blockage and chemical inhibition of sperm motility in the vas deferens by sequential injection of calcium alginate hydrogel, PEG-Au nanoparticles (PEG-AuNps), and ethylene diamine tetraacetic acid (EDTA) (Fig. 11) [29]. Sodium alginate, a natural macromolecular material with excellent biocompatibility, can be guided by GDL to form an injectable system with calcium-containing substances [28]. Calcium alginate hydrogel is considered a long-term physical barrier for sperm and retains its good biocompatibility properties. EDTA can not only be used as a calcium chelator in the hydrogel but also inhibit sperm activity [146]. Importantly, PEG-AuNps is regarded as a temperature-increasing agent which can also be solidified at 37 °C to block both ends of EDTA separately. With sequential injections, sodium alginate was cross-linked to form calcium alginate aqueous solids, which acted as a long-term physical barrier, and PEG-AuNps also sealed both ends of the device by curing. When there was a need for fertility recovery, PEG-AuNps was melted after near-infrared light irradiation, allowing EDTA to come into contact with PEG enabling the vas deferens to be unblocked. The contraceptive period can be set directly by adjusting the injection proportion of each reagent in the range of 2–20 weeks. The embolic area can be easily dredged by a short period of non-invasive near-infrared irradiation rather than a second open surgery.

#### Photothermal nano contraception

The appropriate temperature for sperm formation is slightly lower than the human body temperature. And as the temperature around the testicles increases, sperm production can be disturbed or even stage infertility occurs [147]. Inspired by this theory, an alternative male contraceptive method was developed, which is to inhibit the production of sperm through the photothermal effect of the biomaterial. Li et al*.* proposed an in situ testicular injection of methoxy poly(ethylene glycol)-modified gold nanorods combined with near-infrared light irradiation to achieve medium to long-term male contraception [148]. As a medical biomaterial approved by the U.S. FDA, gold nanoparticles have been widely used in the fields of drug and gene carriers, diagnostics, imaging, etc. with the superiorities of non-toxicity, good stability, fast metabolism, photothermal effect, antibacterial and antioxidant properties [149–151]. In particular, the photothermal effect of gold nanorods has been widely used in the diagnosis and treatment of cancer [152–154]. The PEG coating is designed primarily to evade the immune system [155]. It was demonstrated that the heat treatment not exceeding 40 °C with infrared laser irradiation at 808 nm, the morphology of the testes and vas deferens of male rats was partially damaged and the fertility index dropped to ~ 10% on day 7 and recovered to 50% on day 60. In heat treatment at 45 °C, the morphology of the testes and vas deferens was destroyed and the fertility index dropped to 0 on day 7.

However, in situ testicular injection and heat therapy have reduced the patient's acceptance and limited the popularization of this method of contraception. Another potential magnetic hyperthermia treatment with testis targeting and magnetic hyperthermia for male contraception would be described in the next section.

### Intravenous administration

In comparison to other contraceptive routes, intravenous injections have their unique advantages as well as drawbacks. On the one hand, the circulatory system facilitates the diffusion and immune activation of the biomaterial or drug delivery, making it suitable for immuno-contraception, while the intravenous method is well accepted by patients and easy to administer. On the other hand, contraceptive methods that do not aim to activate the immune system require special attention to avoid immune recognition when administered intravenously, and the metabolic pathway and retention time of the material or drug in the body is still not negligible.

#### Immunocontraception

In the 1930s, it was reported that the foundation of an active immune state by antigens in human semen might induce female infertility [156]. Immunocontraception has been considered a safe, long-term effective, and reversible method with its main targets of action being sperm–egg interaction and maternal recognition of the fertilized egg [157]. Among them, the main challenge comes from the safety, reliability, and reversibility of active immunity to the reproductive system [157]. Chua et al*.* demonstrated a chitosan-based nanoplatform for efficient delivery and immune system uptake of luteinizing hormone-releasing hormone (LHRH) [158]. Chitosan-based nanoparticles modified by functional chloroacetyl groups are allowed to covalently attach the peptide-based and protein-based antigen, which was induced high levels of LHRH-specific antibodies. Meanwhile, nanometer-sized particles are more likely to be uptaken by dendritic cells and retained in lymph nodes than micron-sized particles. After 22 days of inoculation of mice with chitosan-based nanoparticles, the levels of LHRH antibodies were similar to those produced after inoculation with antigen-emulsified complete Freund’s adjuvant, demonstrating the contraceptive effectiveness of chitosan-based nanoparticles. D’Souza et al*.* proposed polyethylene sebacate (PES) encapsulated Sperm-specific 80 kDa Human Sperm Antigen (80 kDa HSA) dissolved in *N*-methyl-2-pyrrolidone as an alternative to Freund’s adjuvant for peptide vaccines [159]. PES is recognized as a stable degradable polymer with good biocompatibility, non-genotoxic, non-mutagenic properties [160]. And compared with the LHRH antibody, the use of the synthetic peptide HSA not only eliminated the need for antigens from biological sources, but also induced a more specific immune response [161]. The formulation formed PES-based nanoparticles of approximately 100 nm in situ after intradermal injection and achieved an antibody titer up to 1:3200. The loss of sperm motility and head-to-head sperm agglutination was observed in the rabbit, and no severe reaction was detected after treatment. It can be seen that the utilization of nanobiomaterials in immunocontraception significantly improves the safety and efficacy of contraception and reduces the side effects caused by pure immunoadjuvants. However, the irreversibility of immunocontraception cannot be effectively verified, which may make its use cautious in younger age groups.

Elevated serum concentrations of interleukins (e.g. IL-6, IL-1β) can promote embryo implantation in the early phase of pregnancy [162]. Based on this, a down-regulator of interleukin which complexed by nano Piperolactam A (nano PLA)-2-hydroxy-propyl-β-cyclodextrin (HPBCD) was proposed [163]. Compared to natural cyclodextrins, the hydroxyl groups of HPBCD are hydroxypropylated, reducing the strong crystallinity while obtaining a better complexation ability. In addition, the hydrophobic core of HPBCD allows hydrophobic molecules to be trapped in it to achieve sustained drug release [164]. Nano PLA was selected for the down-regulation of interleukin secretion as a new hydrophobic anti-fertility drug of natural origin [165]. Serum levels of IL-6 and IL-1β were not increased in pregnant rats after nano PLA-HPBCD manipulation, while estrogen and progesterone secretion was not affected. In addition, no acute or chronic toxicity was observed during the treatment, indicating the nano PLA-HPBCD was expected to be a nature-derived down-regulator of interleukin for female contraception.

Under normal sexual circumstances, immunocontraception is effective in avoiding pregnancy by preventing gamete production and fertilization by the immune system. However, immunocontraception by suppressing relevant immune targets may increase the probability of autoimmune diseases, which may cause ovarian-related diseases and fail to restore fertility. Nanomaterials are promising for safer, time-controlled contraception through targeted transport of proteins or peptides through the circulatory system to their target sites by embedding, chemical modification, and masking other non-relevant immune targets, avoiding the need to stimulate a systemic immune response [166].

#### Magnetic targeting and hyperthermia

**Fig. 12:**
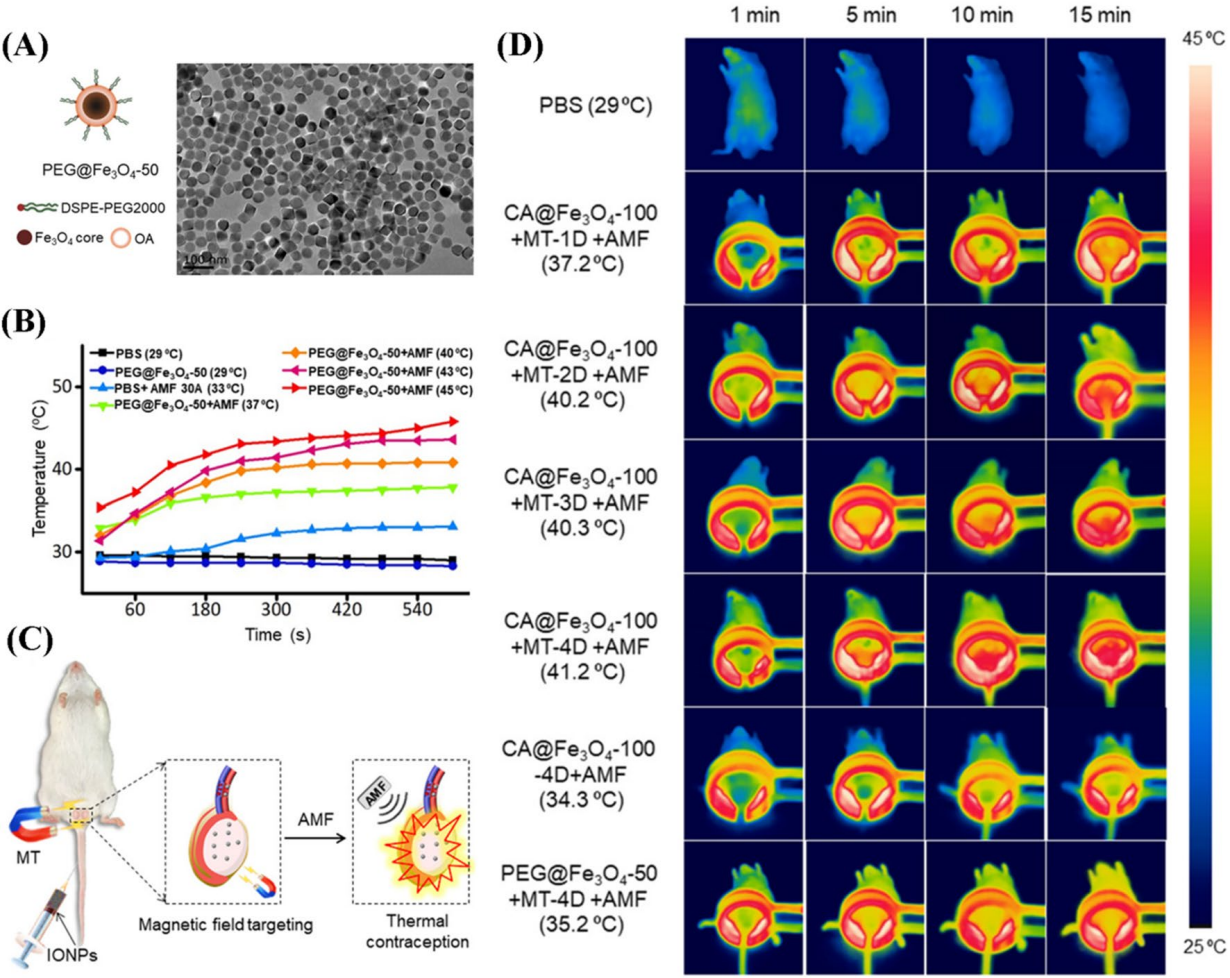
**A** Schematic representation of the design and SEM image of PEG@Fe_3_O_4_-50. **B** The testicular temperature under IONPs thermomagnetic therapy in vivo. **C** Schematic diagram of non-invasive contraception by intravenous injection of PEG@Fe_3_O_4_-50. **D** Thermal infrared images of treated mice under an alternating magnetic field (Reprinted with permission from reference [23]. Copyright 2021 American Chemical Society)

As mentioned above, the severe pain from in situ testicular injection and dermal damage caused by near-infrared light irradiation limited the clinical application of photothermal male contraception [167]. Therefore, the nanotechnology of biomaterials for male contraception also requires optimizing the route of operation and avoiding secondary damage. Iron oxide nanoparticles (IONPs) can be used as potential alternative medical nanoparticles due to their biocompatibility, biodegradability, and physiological metabolic pathways [168, 169]. The unique magnetic therapeutic and targeting properties have been widely used in cancer therapy and drug carrier platforms [170–172]. Ding et al*.* proposed controllable male contraception via intravenous administration. PEG@Fe_3_O_4_ was synthesized by coating oleic acid through the thermal decomposition method (Fig. 12) [23]. 50 nm of Fe_3_O_4_ was selected for exhibiting the best thermal effect on the inhibition of spermatogenesis. After intravenous injection, the nanoparticles were aggregated in the mice testis under the alternating magnetic field while being able to produce a local 37–45 °C environment, effectively reducing the fertility index. Besides, mild magnetic hyperthermia could achieve fertility recovery after 60 days. Compared to direct testicular injection, intravenous injection of nanoparticles would be more acceptable for patients. Importantly, the application of alternating magnetic fields can effectively avoid secondary damage to the skin caused by near-infrared light. Therefore, the combination of targeted delivery with hyperthermia has the potential to enable the clinical translation and application of nanomaterials for contraception and reversible recovery.

## Conclusion and outlook

Following the people’s wishes and usage scenarios, an increasing number of long-term reversible contraceptive options and products have been developed. However, the quest for significant contraceptive effectiveness, fewer side effects, and enjoyable sexual experiences during sex still need to be improved. The development of biomaterials for contraceptive technology can not only effectively improve the initial explosive release of drugs and avoid tissue damage and necrosis, but also take advantage of the excellent properties of the materials themselves to achieve effective birth control in a non-hormonal manner. In addition, with the introduction of modified biomaterials and condition-sensitive materials, it is expected that the need for reversible contraception can be further met by a variety of contraceptive methods while ensuring the effectiveness of contraception.

However, to accelerate the translation of biomaterials-based long-acting reversible contraception from the bench to the clinic, more studies are needed to be performed. The development of various types of biomaterials over the past two decades has improved the inferiority of traditional contraceptive methods, with studies showing more effective drug delivery, more stable physical barrier effects, easier reversible recovery, and less surgical damage. However, the need for personalized contraception may be the next research priority. According to the new protocol, some individualized requirements will be able to be significantly enhanced through the introduction of biomaterials with structural optimization in terms of release control, avoidance of hormonal side effects, and conditional degradation. And there may be in short described as follows.

Although there is a significant growth in the use of nanomaterials in contraception through the advantages of drug delivery and controlled drug release, the side effects of nanotoxicity on the human body cannot be ignored. For example, Cu-IUD, which is commonly used in contraception, is absorbed into the bloodstream through local mucosal tissues and induces hepatotoxicity and nephrotoxicity through mitochondrial failure, enhanced ketogenesis, fatty acid beta-oxidation, and glycolysis, resulting in scattered necrosis of hepatocytes and extensive necrosis of the proximal tubules of the kidney [173]. The second factor lies in the small size and surface charge of the nanomaterials, which can pass through the biological barrier, which reduces glutathione levels, inhibits catalase and superoxide dismutase activities to affect reproductive organs adversely [174]. The impacts of the mode of entry of nanomaterials into the body and the structural changes that occur in the body cycle on their biological effects also deserve to be investigated. In addition, nanomaterials can be recognized by the human immune system as foreign antigens causing unpredictable immune attacks and even the departure of autoimmune diseases. In particular, the particle size, surface morphology, and charge of the material play a crucial role in immunotoxicity and may cause reactive oxygen species production, DNA damage, lysosomal damage, mitochondrial dysfunction, and apoptosis or necrosis [175].

Meanwhile, it is still to be solved that the obstruction can be easily expelled by the peristaltic movement of the muscular duct, which affects the contraceptive effect. Advances in research on structural combinations of biomaterials may improve this deficiency. Structural biomaterial refers to the hierarchical assembly of multiple structural forms of biomaterials for a specific purpose, which makes better use of the advantages among components. As mentioned before, the research about ethylene–vinyl alcohol copolymer and SMA as contraceptive blocker have been made great progress [122, 176]. And on the other hand, scaffolds acted as the support that was constructed from electrospun polycaprolactone and collagen have been shown to perform well in mechanical properties such as tensile strength, maximum load, Young’s modulus, and elongation, which supposed that the tubular scaffold made of biomaterials may be the key point to solve problems [177]. Therefore, a structural biomaterial scaffold might withstand the extrusion of the muscular layer of the pipe preventing the get-away of inner blockages, in addition to protecting the microenvironment within the pipe to attain reversible contraception [178, 179].

## Data Availability

Not applicable.

## Abbreviations

WHO: World Health Organization
Cu: Copper
Cu^2^^+^: Copper ion
Cu-IUD: Copper-containing IUD
PE: Polyethylene
IUD: Intrauterine device
SMA: Styrene maleic anhydride
PLGA: Polylactic acid co-glycolic acid
GDL: Gluconolactone
CS: Chitosan
β-GP: Beta-sodium glycerophosphate
GnRH: Gonadotropin-releasing hormone
LH: Luteinizing hormone
FSH: Follicle-stimulating hormone
MIS: Mullerian inhibiting substance
hCG: Human chorionic gonadotropin
E2: Estradiol
hCG: Human chorionic gonadotropin
DMPA: Depot medroxyprogesterone acetate
Hormonal-IUD: Hormonal intrauterine device
LNG: Levonorgestrel
EVA: Ethylenevinglacetate
POE: Poly (ortho esters)
NET: Norethisterone
PLA: Polylactic acid
PGA: Polyglycolic acid
PCL: Poly-(ε-caprolactone)
FDA: U.S. Food and Drug Administration
DMN: Dissolving microneedle
HP-β-CD: Hydroxypropyl beta cyclodextrin
PLGA: Polylactic acid co-glycolic acid
SEM: Scanning electron microscopy
PVP: Polyvinylpyrrolidone
PDMS: Polydimethylsiloxane
MPA: Medroxyprogesterone acetate
PEVA: Polyethylene vinyl acetate
MTT: Montmorillonite
IUB: The intrauterine ball
Mg: Magnesium
UFG-Cu: Ultra-fine grained bulk Cu
PVA: Polyvinyl alcohol
IDM: Indomethacin
PF: Polyols
BZK: Benzalkonium chloride
EVOH: Ethylene–vinyl alcohol
DMSO: Dimethyl sulfoxide
IVD: Intra-vas device
PEG-AuNps: PEG-Au nanoparticles
EDTA: Ethylene diamine tetraacetic acid
LHRH: Luteinizing hormone-releasing hormone
PES: Polyethylene sebacate
IONPs: Iron oxide nanoparticles
